# Comparative transcriptome analysis reveals heat stress-responsive genes and their signalling pathways in lilies (*Lilium longiflorum* vs. *Lilium distichum*)

**DOI:** 10.1371/journal.pone.0239605

**Published:** 2020-10-02

**Authors:** Yongyao Fu, Liping Yang, Haihong Gao, Xu Wenji, Qiang Li, Hongqun Li, Jian Gao

**Affiliations:** 1 School of Advanced Agriculture and Bioengineering, Yangtze Normal University, Fuling, Chongqing, China; 2 Citrus Research Institute, Southwest University/Chinese Academy of Agricultural Sciences, Chongqing, China; Youngstown State University, UNITED STATES

## Abstract

The lily, a famous bulbous flower, is seriously affected by high temperatures, which affect their growth and production. To date, the signalling pathways and the molecular mechanisms related to heat response in *Lilium* have not been elucidated. In this study, a comparative transcriptome analysis was performed in an important thermo-tolerant flower, *L*. *longiflorum*, and a thermo-sensitive flower, *L*. *distichum*. Lily seedlings were first exposed to heat stress at 42°C for different lengths of time, and the optimal time-points (2 h and 24 h) were selected for RNA sequencing (RNA-seq). Approximately 66.51, 66.21, and 65.36 Mb clean reads were identified from three libraries of *L*. *longiflorum* (LL_CK, LL_T2h and LL_T24h, respectively) and 66.18, 66.03, and 65.16 Mb clean reads were obtained from three libraries of *L*. *distichum* (LD_CK, LD_T2h and LD_T24h, respectively) after rRNA removing. A total of 34,301 unigenes showed similarity to known proteins in the database NCBI non-redundant protein (NR), Swiss-Prot proteins, InterPro proteins, Clusters of Orthologous Groups (COG) and Kyoto Encyclopedia of Genes and Genomes (KEGG). In addition, 1,621 genes were differentially expressed in the overlapping libraries between LL_DEGs and LD_DEGs; of these genes, 352 DEGs were obviously upregulated in *L*. *longiflorum* and downregulated in *L*. *distichum* during heat stress, including 4-coumarate, CoA ligase (*4CL*), caffeoyl-CoA O-methyltransferase (*CCoAOMT*), peroxidase, pathogenesis-related protein 10 family genes (*PR10s*), 14-3-3 protein, leucine-rich repeat receptor-like protein kinase, and glycine-rich cell wall structural protein-like. These genes were mainly involved in metabolic pathways, phenylpropanoid biosynthesis, plant-pathogen interactions, plant hormone signal transduction, and kinase signalling pathways. Quantitative RT-PCR was performed to validate the expression profiling of these DEGs in RNA-seq data. Taken together, the results obtained in the present study provide a comprehensive sequence resource for the discovery of heat-resistance genes and reveal potential key components that are responsive to heat stress in lilies, which may help to elucidate the heat signal transcription networks and facilitate heat-resistance breeding in lily.

## Introduction

High temperature is a significant environmental factor that affects plant growth and development. Heat stress, triggered by high temperatures, causes a set of physiological and biochemical changes in plants, such as the inhibition of photosynthesis and damage to the cell membrane, thereby leading to cell senescence and even cell death [1]. In addition, when exposed to high temperature, plants can display growth stagnation, the abortion of flower buds and a decline in economic yield [2, 3]. It is concerning that as global temperatures continue to increase, extreme heat events may threaten food and environment safety in the 21^st^ century [4]. Thus, it is of great urgency to discover the genes related to heat tolerance and investigate the defence mechanisms against heat stress.

As sessile organisms, plants have evolved numerous mechanisms to adapt to high temperature stress, including long-term evolutionary phenological and morphological adaptations and short-term avoidance/acclimation mechanisms. In past decades, major progress has been made in understanding plant response to heat stress. Various signal transduction components, such as phytohormones, sugar (as a signalling molecule), Ca-dependent protein kinases (*CDPKs*), and mitogen-activated protein kinase (*MAPK/MPKs*), are closely associated to the activation of heat-responsive signalling pathways [5]. Some key factors are ion transporters, osmoprotectants, antioxidant proteins and transcription factors (TFs), which have been demonstrated to be involved in the regulation of the plant response to high temperatures [2, 3, 6]. Transcription profiling of thermo tolerant and/or thermo susceptible genotypes has shown that many genes are influenced by high temperatures, including heat stress transcription factors (*HSFs*), heat shock proteins (*HSPs*), calmodulin CaM3 and genes involved in membrane fluidity, chromatin changes, RNA unfolding, enzymatic reactions, such as reactive oxygen species (ROS), and programmed cell death [7]. Furthermore, the upregulation of several genes has been reported to enhance plant tolerance to high temperatures. These genes include Calmodulin-binding protein phosphatase *PP7* [8], protein phosphatase *RCF2* and *NAC19* [9], HEAT-INDUCED TAS1 TARGET1 (*HTT1*) in *Arabidopsis* [10] and SUMO E3 Ligase *SlSIZ1* in tomato [11], in addition to the numbers of *HSFs* that act as key transcription switches in regulating the activation of genes responsive to heat stress in virous plants [12].

The lily, known for the attractive variations in colours, patterns and shapes, is one of the best cut and potted flowers and the lily bulb is always used as a vegetable for editable or medicinal in china, such as *Lilium lancifolium* thumb., *L*. *brownii* var.viridulum and *L*. *davidii* var. unicolor. However, the lily mainly enjoys cool-humid climate conditions, the scale of temperature is at approximately 18–24°C. Exposure to high temperature can result in the wilting of leaf tissues, abortion of flower buds, a reduction in cut flowers and even the degeneration of bulbs [13–15]. In most places in China, high temperatures can exceed 37°C, and the maximum can reach 45°C in the summer, becoming a barrier to lily production throughout the year. Therefore, it is very important to enhance the thermo-tolerance of lily plants by breeding heat-resistant lily cultivars.

In recent years, increasing attention has been paid to the investigation of the mechanisms of thermo-tolerance in lily plants. *HsfA2* was first cloned from *Lilium longiflorum*, and overexpression of *LlHsfA2* enhanced the heat tolerance of transgenic *Arabidopsis* plants [14]. *LlHsfA1* interacting with *LlHSFA2* played important roles in the heat stress response of lily [16]. A small heat shock protein named *HSP16*.*45*, based on the molecular weight of its protein, was cloned from *L*. *davidii*, and the heterologous expression of *LimHSP16*.*45* in *E*. *coli* or *Arabidopsis* further improved cell viability under extreme temperatures [17, 18]. In addition, *LlCaM3* expression was induced by heat and CaCl_2_ in lily, and its overexpression increased the transcript levels of *HsfA1a* and *Hsp18*.*2* and improved the thermo-tolerance of transgenic plants, suggesting that *LlCaM3* is a key component in the Ca^2+^-CaM HS signalling pathway in lily [15]. However, traditional cloning and genetic transformation methods are difficult and time-consuming for further study of the mechanisms of thermo-tolerance.

Transcriptome changes in response to heat stress as well as combined stresses have been reported in several plant species, including *Arabidopsis thaliana* [19], *Solanum lycopersicum* [20], Chinese cabbage [21], Wheat [22], Carnation [23] and *Pinus radiata* [24], etc. However, fewer studies have focused on heat stress-related metabolic pathways and the molecular signalling network in lily. The wild lily *L*. *longiflorum* is well-known for its strong thermo-tolerance [13], while *L*. *distichum* is a thermo-sensitive species only found in Korea and northeastern China [25]. In the present study, the physiological and biochemical changes of these two lily species with different thermo-tolerance levels were determined in response to short-term heat (42°C) stress. Three RNA libraries of *L*. *longiflorum* leaves in contrast to that of *L*. *distichum* leaves were produced using the RNA-seq method. The results revealed that 1,621 common unigenes were significantly responsive to heat stress, of which 350 unigenes were upregulated in *L*. *longiflorum* but downregulated in *L*. *distichum*. In contrast, 133 unigenes showed decreased levels in *L*. *longiflorum* but increased abundance in *L*. *distichum*. These unigenes were composed of glycine-rich cell wall structural protein-like, leucine-rich repeat receptor-like protein kinase (*IMK2* and *IMK3*), 4-coumarate-coA ligase (*4CL*), caffeoyl-CoA O-methyltransferase-like (*CCoAOMT*), protein G1-like 1, glutathione transferase GST 23-like, glutathione S-transferase U17/U18-like, peroxidase (*FLXPER3*) and cationic peroxidase 1/SPC4-like, etc. The collected data provide new insights into heat tolerance in lily plants and contribute to the development of heat-tolerant plants through genetic modification approaches.

## Materials and methods

### Plant material and experimental design

The heat-tolerant *L*. *longiflorum* and the heat-sensitive *L*. *distichum* were selected and cultivated in the tissue culture room of Yangtze Normal University under the conditions of 70% relative humidity under 25°C/18°C (day/night) and 16 h/8 h (light/dark). After 2 months of growth in aseptic condition from lily scales, the lily plantlets were comprised two groups. The control sample (CK: 0 h, 2 h, 8 h, 16 h, 24 h, 48 h, 72 h) was exposed to 25°C in 16 h/8 h (light/dark) and the heat-treated samples (HT: treatment for 0 h, 2 h, 8 h, 16 h, 24 h, 48 h, 72 h) was exposed to 42°C in the same light/dark conditions in a climate chamber respectively. At each time point, three bottles of these lily samples, each with 4 or 5 independent seedlingswere cleaned and immediately frozen in liquid nitrogen for RNA extraction and the same sampling were further performed for biochemical analysis. We abstracted cDNAs from LD_CK(0h), LD_CK(2h), LD_CK(24h), LD_0h, LD_2h, LD_24h, and LL_CK(0h), LL_CK(2h), LL_CK(24h), LL_0h, LL_2h and LL_24h, a total of 36 samples for qRT-PCR experiment with three replicates. Three independent biological replicates were performed, and a total of 42 samples for each type of lily were prepared together with the control sample, which were selected for physiological analysis of chlorophyll, electrical conductivity, carbohydrate and proline analyse.

### Physiological analysis of chlorophyll, electrical conductivity, carbohydrate and proline

Total chlorophyll content was quantified according to the method described by Sims and Gamon (2002) in three replicates per treatment [26]. Pigments were extracted with 10 mL of acetone/Tris (50 mM) buffer at pH 7.8 (4:1) (v/v). After homogenization and centrifugation, supernatants were used to read absorbance at 537, 647 and 663 nm (UV-1800, www.aoe-sh.com, Shanghai, China), and the pigment content was determined.

Electrical conductivity was measured using an electrical conductivity analyser (DDS-307, mL.fzchina.com, Chengdu, China). Three replicates of 100 mg of fresh leaves were collected after high temperature exposure and immediately immersed in sterile de-ionized water. After incubation at room temperature in 24 mL under agitation at 40 rpm, the initial conductivity of each was recorded as S1. Then, the leaves were cooked in boiling water for 15 min; after cooling to room temperature, the total conductivity of each sample was recorded as S2. Relative electrical conductivity (REC) was calculated using the following equation:

REC(%)=(S1/S2)×100.

Data of the total soluble sugars and the starch content were collected as previously described in Escandon *et al*. (2016) [24]. Briefly, 50 mg of each frozen leaf was used to extract total soluble sugars, and after centrifugation, the pellet was used to quantify starch content. Absorbance was read at 625 nm and both contents were determined against a D-glucose standard curve. Three biological replicates were performed for each time-point treatment.

Proline content was determined according to Bates *et al*. (1973) [27]. Three replicates of 100 mg of frozen leaves were homogenized in 4 mL of 3% sulfosalicylic acid and centrifuged. Equal volumes (2 mL) of ninhydrin, glacial acetic acids and the supernatant were incubated at 100°C for 1 min. After cooling to room temperature, 4 mL of toluene was added for the extraction of the chromophore, and the absorbance was analysed at 520 nm by spectrophotometer (UV-1800, www.aoe-sh.com, Shanghai, China). A standard curve of proline was used for calculating the proline concentration.

### RNA isolation sequencing library construction and sequencing

According to the manufacturer’s instructions, total RNA was extracted from plantlets using TRIzol Reagent (Invitrogen, USA), and then contaminated DNA was removed from the extracted RNA by treated with DNase I (Promega, USA). The RNA quality and purity were verified using Nanodrop 2000 and an Agilent 2100 Bioanalyzer (Santa Clara, CA, USA) respectively. Then, high-quality RNA samples of each time point in three independent biological replicates were mixed and then used for RNA sequencing. The quality assessment should yield an OD_260/280_ ratio of 1.8 to 2.0 and an OD_260/230_ ratio of 2.0 to 2.2 with latter being an additional value for purity determination. A total of 6 cDNA libraries were constructed, comprising three libraries of *L*. *longiflorum* (LL_CK, LL_T2h, and LL_T24h) and three libraries of *L*. *distichum* (LD_CK, LD_ T2h, and LD_ T24h), using the BGISEQ-500 platform at the Wuhan, Hubei Province (BGI’ Tech, China). In brief, the total RNA was purified to enrich poly(A) mRNA with magnetic oligo (dT) beads. The target RNA was fragmented and then used for dscDNA library construction by random hexamer (N6) primers. The ends of the dscDNA were repaired with phosphate at the 5’ end and sticky ‘A’ at the 3’ end, after which the dscDNA strands were ligated with adapters that had a sticky ‘T’ at the 3’ end. Two specific primers were used to amplify the ligation product. Finally, the PCR product was denatured by heat, and the single-strand DNA was cyclized by splint oligo and DNA ligase. Six cDNA libraries were constructed and sequenced on a BGISEQ-500 RS platform at BGI. All raw read sequences are available in the NCBI sequence read archive under the accession number PRJNA577293 and the assembled transcript sequences were also submitted to the NCBI TSA database (GIUQ00000000).

### Reads processing and identification of differentially expressed genes (DEGs)

Clean reads were obtained by filtering out adaptor-only reads, trimming reads containing more than 5% unknown nucleotides, and low-quality reads with the percentage of low quality bases (base quality ≤10) using Trimmomatic and we subsquently aligned the clean high-quality reads to the SSU and LSU rRNA sequences using BWA software. After that, we assembled the clean reads from the heat-tolerant *L*. *longiflorum* after rRNA removing using de novo assembly program Trinity except K-mer value to conduct the de novo assembly [28]. Additionally, only one read copy will be kept for assembly and redundant duplication reads be eliminated for mutli-duplication’s reads, After that, overlapped nucleic acid sequence were generated to the contigs assembled using Trinity. To obtain the unigene, the paired-end reads were used for constructing scaffolds with the paired end information by realigning to contigs. Then, these contigs in one transcript were assembled by the Trinity and gained the sequence not being extended on either end defined as unigenes. To harvest as much description as possible for the assembled sequences, all unigenes were annotated based on BLAST searches using BLASTx search tool through Swiss-Prot protein databases and the National Center for Biotechnology Information non-redundant protein (Nr) with threshold E-value set as less than 1e-10, identity > 70%, query coverage ≥ 80%, and other parameters were defaulted. In general, we used BLAST alignment of transcripts (or translations of predicted ORFs from transcripts) to reference protein sets as a means of assessing coding transcript completeness. Transcripts with ≥ 80% sequence coverage (i.e. a significant alignment between a transcript sequence from our assembly and a target protein sequence, where the alignment covers at least 80% of the target protein sequence) are thus considered “full or near-full length”. In addition, the conserved ortholog content was identified using the nematod Benchmark Universal SingleCopy Orthologs (BUSCOs, v4.0.6). To further predict their functions, based on Nr and SwissPro BLAST results, the unigenes were then annotated in Kyoto Encyclopedia of Genes and Genomes (KEGG, http://www.kegg.jp/) database and Gene Ontology (GO, http://www.geneontology.org/) database. For the nr annotations, the BLAST2GO program was used to assign GO annotations (comprised of biological processes, molecular functions, and cellular components) of unique assembled transcripts [29]. Subsequently, WEGO (Web Gene Ontology Annotation Plot) software was used to conduct GO functional classification for understanding the distribution of gene functions at the macroscopic level [30]. After that, Bowtie2 was adopted to map the clean reads to the de novo assembly transcriptome reference sequences, and based on the mapping of RNA-seq reads to the assembled transcriptome, the developed software RSEM was performed to assess transcript abundances by quantification of the de novo assembly transcript and calculated as the FPKM (fragments per kilobase of transcript per million mapped reads); Expression data from two libraries (treatment and control) were determined by mapping to the transcriptome assembly using Bowtie2 software [31]. The fragments per kilobase of transcripts per million fragments mapped (FPKM) values were analysed further using RESM [32] and PossionDis [33] to get differentially expressed genes (DEGs) between the control and infected groups [(LL_T24h vs. LL_CK and LL_T2h vs. LL_CK for *L*. *longiflorum*) and (LD_T24h vs. LD_CK and LD_T2h vs. LD_CK for *L*. *distichum*)]. Further, to determine the threshold *p*-value in multiple tests, a false discovery rate (FDR) was used. Furthermore, significant enrichment was calculated when FDR was <0.05 and FPKM values showed at least a two-fold difference between the two samples reads. Furthermore, DEGs related to heat-stress responsiveness were analysed and plotted using Neighbour–Joining cluster through homemade R script. Followed by the coefficient of variation with a threshold of 1 across the different samples, these genes were selected and then transformed FPKM values by log2.

### GO functional annotation and KEGG pathway analysis of common differently expressed genes (DEGs) in lilies

Common DEGs in both lily species responsive to heat stress were identified using the venny program (https://bioinfogp.cnb.csic.es/tools/venny/); subsequently, we submitted those common DEGs for GO annotation through Gene Ontology Database (http://www.geneontology.org/) and KEGG pathway enrichment analysis using KOBAS software. GO functional categories were assigned to differentially expressed genes based on Gene Ontology Database (http://mL.geneontology.org/); the false discovery rate (FDR) was calculated to correct the P-value for the GOs of all the DEGs. Moreover, KOBAS software [34] was used for pathway enrichment analysis by testing the statistical enrichment of DEGs in the KEGG pathway; the common DEGs in *L*. *longiflorum* (LL_T24h vs. LL_CK and LL_T2h vs. LL_CK) and *L*. *distichum* (LD_T24h vs. LD_CK and LD_T2h vs. LD_CK) were annotated to the KEGG database, which exhibited all significant enrichments for DEGs in the pathway. A P-value and FDR (FDR < 0.05) were presented after pathway analysis for each type. After that, their relative graphs were constructed using R script.

### Verification of the gene expression profiles of candidate DEGs by qRT-PCR in lilies

Quantitative real-time PCR was performed on QuantStudio Real-time PCR system (Applied Biosystems by Thermo Fisher Scientific) platform using SYBR Premix Ex Taq (Cat. #RR420L, TakaRa, China) following the manufacturer’s instructions. The primers employed in the qRT-PCR experiments are designed by GenScript Molecular Biology Tools online (https://mL.genscript.com/tools/real-time-pcr-tagman-primer-design-tool) (S1 Table). The *SAND* gene was selected as the internal control according to a previous analysis [35]. At least three independent biological replicates of RNA samples at each time point of heat treatment including CK were obtained from these lily plantlets and collected for qRT-PCR analysis. The first-stand cDNA was synthesized using PrimeScript^™^ RT reagent Kit with gDNA Eraser (Cat. #RR047A, TakaRa, China). Amplification was performed using PCR as follows: a first denaturation step at 95°C for 3 min; 40 cycles of denaturation at 95°C for 15 s; followed by annealing and extension at 60°C for 1 min. Melting curves were generated to verify amplification specificity by a thermal denaturing step at 95°C for 15 s and 60°C for 1 min, and then from 60°C to 95°C slowly (0.15°C/S). To calculate the fold change in the expression levels of target genes, the relative quantitative method (2^-ΔΔCT^) was selected and performed with three technical replicates for all reactions.

### Statistical analysis

To evaluate differences between difference samples in *L*. *longiflorum* and *L*. *distichum*, variance analysis was carried out for the Student’s t-test using SPSS statistical software, significant differences among the relative expression levels of the genes were shown to be statistically significant (P<0.05).

## Results

### Physiological changes of lily seedings under heat stress conditions

**Fig 1:**
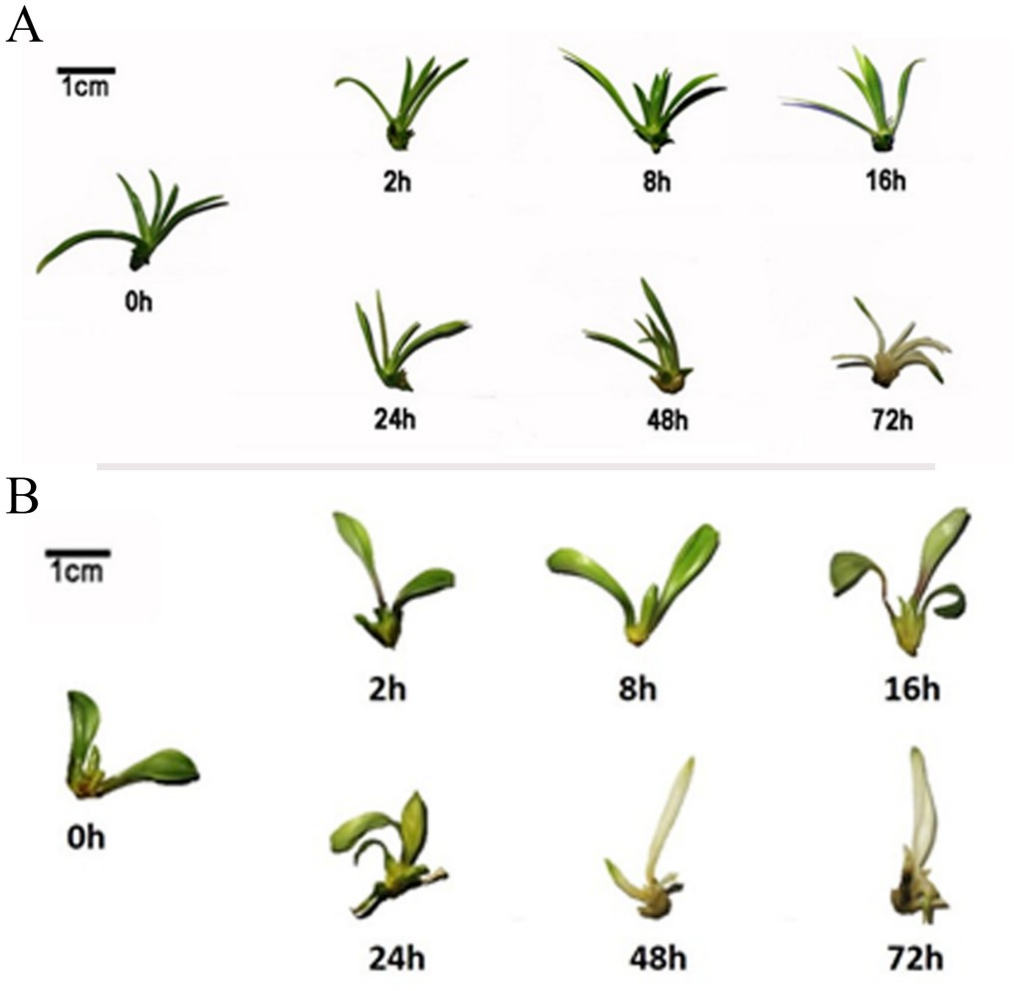
Dynamic changes of growth development for different lilies (*L*. *longiflorum* and *L*. *distichum*) subjected to high temperature (42°C) treatment for six lengths of time (2 h, 8 h, 16 h, 24 h, 48 h and 72 h). Fig 1A. Dynamic changes of growth development for *L*. *longiflorum* under heat stress for different treatment times. Fig 1B. Dynamic changes of growth development for *L*. *distichum* under heat stress for different treatment time points.

**Fig 2:**
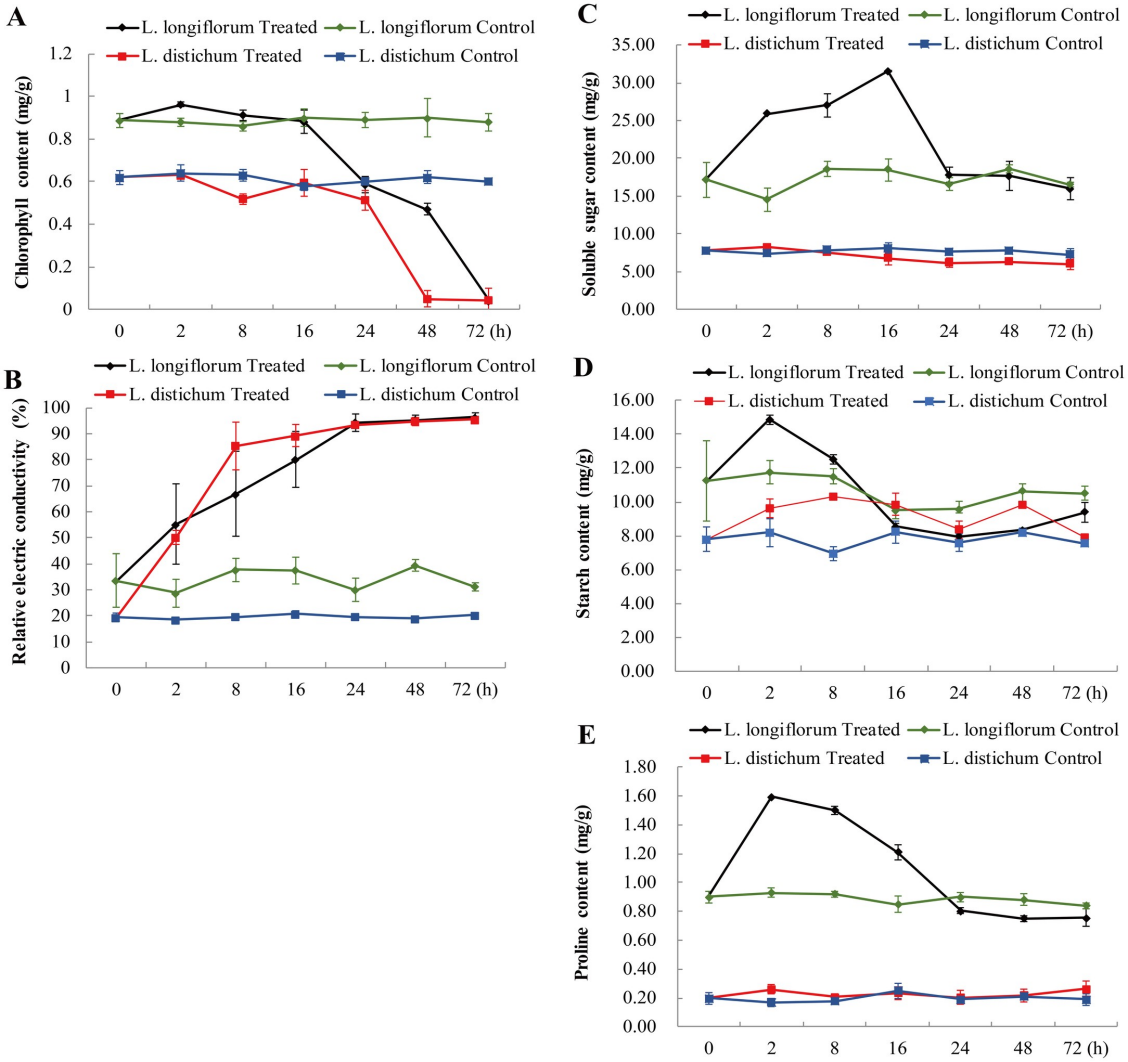
Physiological changes of chlorophyll content, soluble sugar content, relative electric conductivity, starch content and proline content for lily seedings under heat stress conditions. Fig 2A. Dynamics changes of chlorophyll content determined in the heat-tolerance *L*. *longiflorum* and the heat-sensitive *L*. *distichum*. Fig 2B. Dynamics changes of relative electric conductivity validated by the heat-tolerance *L*. *longiflorum* and the heat-sensitive *L*. *distichum*. Fig 2C. Dynamics changes of soluble sugar content identified in the heat-tolerance *L*. *longiflorum* and the heat-sensitive *L*. *distichum*. Fig 2D. Dynamics changes of starch content determined in the heat-tolerance *L*. *longiflorum* and the heat-sensitive *L*. *distichum*. Fig 2E. Dynamics changes of proline content identified in the heat-tolerance *L*. *longiflorum* and the heat-sensitive *L*. *distichum*.

To determine the optimal time to analyse the heat response for lilies (*L*. *longiflorum* and *L*. *distichum*), uniform plantlets with 2 months of growth in aseptic conditions were subjected to high temperature (42°C) treatment and then sampled at six different times of stress (2 h, 8 h, 16 h, 24 h, 48 h and 72 h). As shown in Fig 1, the plantlets of *L*. *longiflorum* grew normally, but the leaves gradually turned white with longer durations of heat stress. After 24 h, the colour of *L*. *longiflorum* leaves started to change but no signs of wilting, and the plantlets lost their green colour during 48 h to 72 h (Fig 1A). By contrast, the leaves of *L*. *distichum* started to wilt accompanied by a colour change after 24 h; the leaves lost almost of their green colour at 48 h and finally appeared to be dying after 72 h (Fig 1B). The analysis of chlorophyll content indicated coincident results with the colour change in lilies (Fig 2). To determine leaf membrane damage, measurements of the electric conductivity showed that the levels were rapidly increased after 2 h in both lilies and reached their peak after 24 h in *L*. *longiflorum* and 8 h in *L*. *distichum* (Fig 2B). Furthermore, the levels of the measured soluble sugar and the starch increased rapidly and sharply following heat exposure of *L*. *longiflorum* leaves compared with *L*. *distichum* leaves; however, the starch level declined faster than that of the soluble sugar (Fig 2C and 2D). The general trends of the soluble sugar and proline were similar; both leveled off at 24 h, although they reached their peaks at different times, with the former reaching its peak at 16 h and the latter at 2 h (Fig 2E). The entire physiological index showed that *L*. *longiflorum* was more responsive to the high temperature condition than *L*. *distichum*, which is consistent with the heat resistance of these two lilies. Taken together, the following experiments of the two lilies were performed at 0 h (CK) and two different heat-treatment times designated as LL_CK, LL_T2h, and LL_T24h for *L*. *longiflorum* in contrast to LD_CK, LD_ T2h, and LD_ T24h for *L*. *distichum*, accounting for the early heat response and the physiological changes.

### Illumina sequencing and de novo assembly of lily transcriptome responding to heat stress

**Fig 3:**
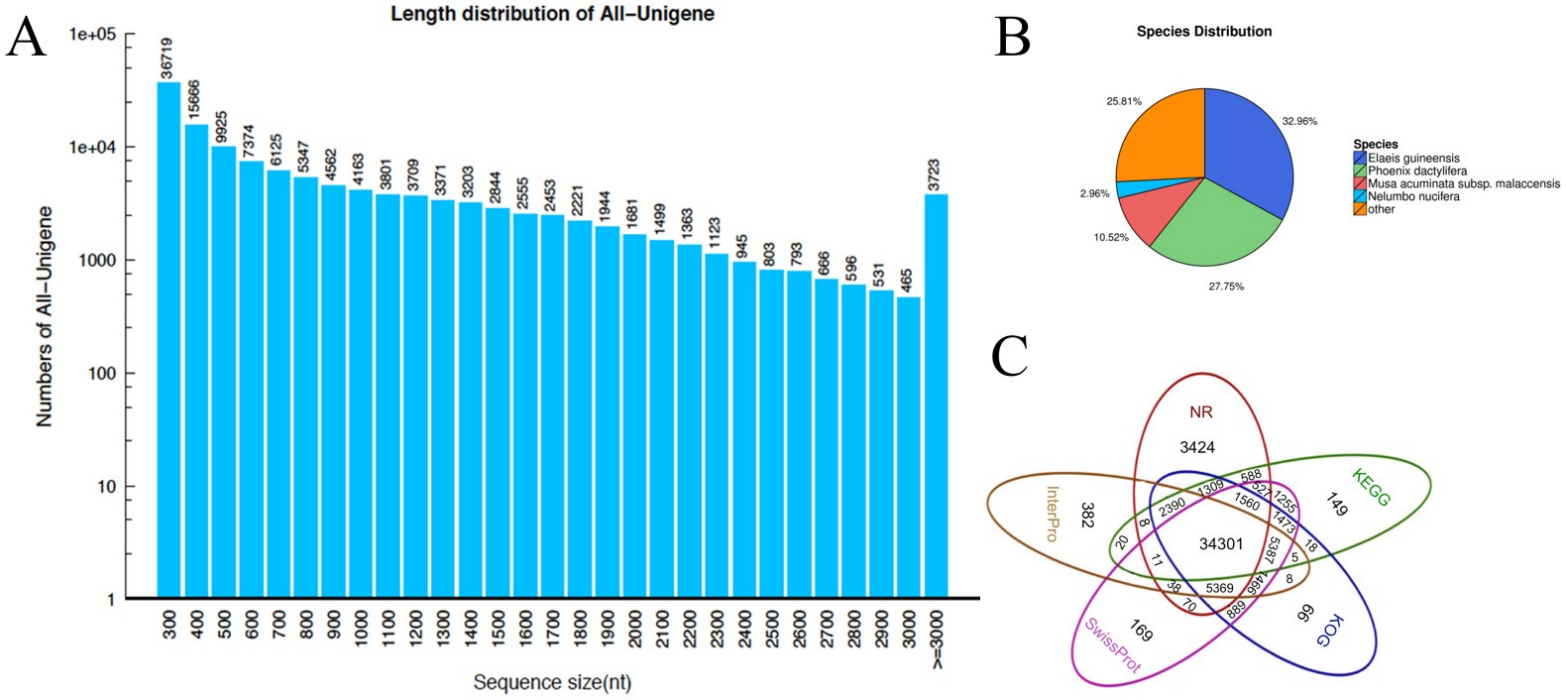
Comprehensive overview of de novo assembly of lily transcriptome responding to heat stress in lily. Fig 3A. length distribution of the assembled contigs and unigenes obtained from de novo assembly of lily transcriptome. Fig 3B. Species distribution of the top BLAST hits for lily assembled transcriptome. Fig 3C. Comprehensive overview of BLASTx alignment against the public protein databases including Nr, KEGG, COG, Swiss-Prot and InterPro proteins.

To develop a comprehensive overview of gene expression profiles of lilies (*lilium sp*.) responding to high temperature stress, three libraries (LL_C, LL_T2h and LL_T24h) of *L*. *longiflorum* in contrast to that (LD_C, LD_T2h and LD_T24h) of *L*. *distichum* were constructed. The RNA samples were prepared from lily fleshy leaf with differential thermotolerance and subjected to pair-end read (PE) sequencing using the BGISEQ-500 platform at Beijing Genomics Institute (BGI, Wuhan, China). PE sequencing could increase the depth of sequencing and improve *de novo* assembly efficiency [36]. As a result, 74.71, 74.71, and 72.22 Mb raw reads were obtained from the LL_C, LL_T2h and LL_T24h libraries, respectively, and 74.71, 74.71, and 74.71 Mb raw reads in LD_C, LD_T2h and LD_T24h libraries, respectively. After removing adaptor sequences and low-quality reads, 66.51, 62.61, and 65.36 Mb clean reads were generated in three libraries of *L*. *longiflorum* and 66.18, 66.03, and 65.16 Mb clean reads in three libraries of *L*. *distichum* (S2 Table). Subsequently, 198, 342 contigs were constructed from the raw sequence reads with an average length of 411 bp, and 130, 170 unigenes with a N50 length of 1442 bp were generated with an average length of 875 bp using paired-end reads in all the libraries. The percent of length distribution of the assembled contigs and unigenes is shown in Fig 3A, which indicated that unigenes mainly ranged from 300 to 3000 bp, and the majority were distributed from 300 to 1800 bp (over 2000 numbers) accounting for more than 69.2% of all of the unigenes. The assembly completeness has been assessed by calculating the BUSCO. The assembled transcripts were compared against the benchmarking universal single-copy orthologs (BUSCO) revealing that almost 65% of the BUSCO groups have complete gene representation, while 10% are only partially recovered, and 25% are missing (S1 Fig). It is probable that the assembly completeness was not good owing to the bigger and complex of the lily genome; there was not currently genome of the lily provided for research. To evaluate completeness of transcriptome libraries and annotations of all the assembled unigenes, the annotated sequences were corresponded to the known nucleotide sequences of plant species, with 32.96%, 27.75%, 10.52%, and 2.96% matching with *Elaeis guineensis*, *Phoenix dactylifera*, *Musa acuminate subsp*. *malaccensis*, and *Nelumbo nucifera* respectively, and the remaining 25.81% matching with others (except for the top matched species) (Fig 3B). All of the top three species with BLAST hits were involved in the Palmaceae family (almost 60.71% of all assembled transcripts) and the Musaceae family (10.52% of all assembled transcripts), implying that the sequences of the lily transcripts aligned more closely to these two families. Through the BLASTx alignment against the public protein databases including Nr, KEGG, COG, Swiss-Prot and InterPro proteins, the results indicated that a total of 34,301 unigenes (89.11% of all unigenes) were annotated and matched to all of the public protein databases (Fig 3C).

### Functional annotation and classification of the assembled unigenes from lily transcriptome

Based on sequence homology, the Gene Ontology (GO) assignments system was performed to classify the roles of these uncharacterized sequences. By using the BLAST2GO program, all assembled unigenes were assigned to three main categories such as “biological process” (BP), “cellular component” (CC) and “molecular function” (MP), with a total of 22,659, 24,799 and 12,212 unigenes, respectively. Then, these annotated sequences were assigned to GO-slim terms to facilitate the functional distribution of plants. All GO terms were subsequently divided into 54 functional groups such as biological processes (23 groups), cellular component (17 groups), and molecular function (14 groups). Within the biological processes, the metabolic process including 5,754 (25.4%) unigenes and cellular process including 5,445 unigenes (24.0%) were the most dominant. For cellular component, the majority of GO terms were primarily assigned to the cell and cell part including 5,103 (20.6%) and 5,055 unigenes (20.4%), respectively. With regards to molecular function, those assignments were focused on catalytic activity including 5,543 unigenes (45.4%) and binding including 5,010 unigenes (41.0%) (S2A Fig).

In addition, blast analysis from the COG database indicated that all the unigenes were assigned to 15 COG categories. Among these categories, the cluster for ‘General functions prediction only’ related to basic physiological and metabolic functions accounted for the largest group, followed by ‘Posttranslational modification, protein turnover, chaperones’, ‘Function unknown’, ‘Defense mechanisms’, ‘Cytoskeleton’, ‘Lipid transport and metabolism’, and ‘Intracellular trafficking, secretion, and vesicular transport’, while fewer unigenes were assigned to ‘Nucleotide transport and metabolism’, ‘Coenzyme transport and metabolism’ and ‘Nuclear structure’ (S2B Fig). These findings revealed that a great deal of genes with molecular functions were related to numerous biological progress under high temperature stress in lilies.

Moreover, the KEGG pathway database contributed to better understand the biological roles of genes involved in different networks [37]. Through comparing against the KEGG pathway database with KEGG Orthology (KO) numbers using BLASTx alignments with a cut-off E value of 10^−5^, there were altogether 51,199 unigenes belong to 21 KEGG pathways annotated and assigned. Of these categories, the most represented pathways were ‘Transport and catabolism’ (2,291, 4.47%), Signal transduction (2,445, 4.78%), Translation (4,065, 7.94%), Carbohydrate metabolism (4,427, 8.65%) and Environment adaption (1,788, 3.49%) (S2C Fig). All of the annotations provide valuable references for inquiring into these processes, pathways and functions involved in the short-term high-temperature response.

### Cluster analysis of gene expression in lilies

To investigate these key candidate genes involved in short-term heat response of lilies, we conducted the RNA-Seq experiments using tangential sections of LL_C, LL_T2h, and LL_T24h of *L*. *longiflorum* and LD_C, LD_T2h and LD_T24h of *L*. *distichum* according to physiological changes and mapped the resulting reads to the reference transcriptome. To minimize false negatives and positives, a statistical analysis was based on unigenes with a FPKM value ≥ 1 in at least one of the three stages (S3 Table). Notably, statistical significance was according to expected sampling distributions. Cluster analysis was performed by the k-means method according to the gene expression dynamics [38]. Differential expression genes were divided into 9 groups associated with their expression profiles (S3 Fig). Transcript levels of these DEGs were significantly altered during three different stages of heat stress in both lilies. Of all the DEGs, a part were downregulated from the 0 h to 2 h timepoint but were upregulated from the 2 h to 24 h timepoint, while another part indicated increased quantitative expression at 2 h but decreased expression at 24 h. To identify genes showing evidently expression changes, during different heat stress stages, these expressed tags with alteration among the three samples of the two lilies were sorted by an algorithm developed from the heat-map (S3 Fig).

### Differential expression genes (DEGs) associated with heat stress in lily

**Fig 4:**
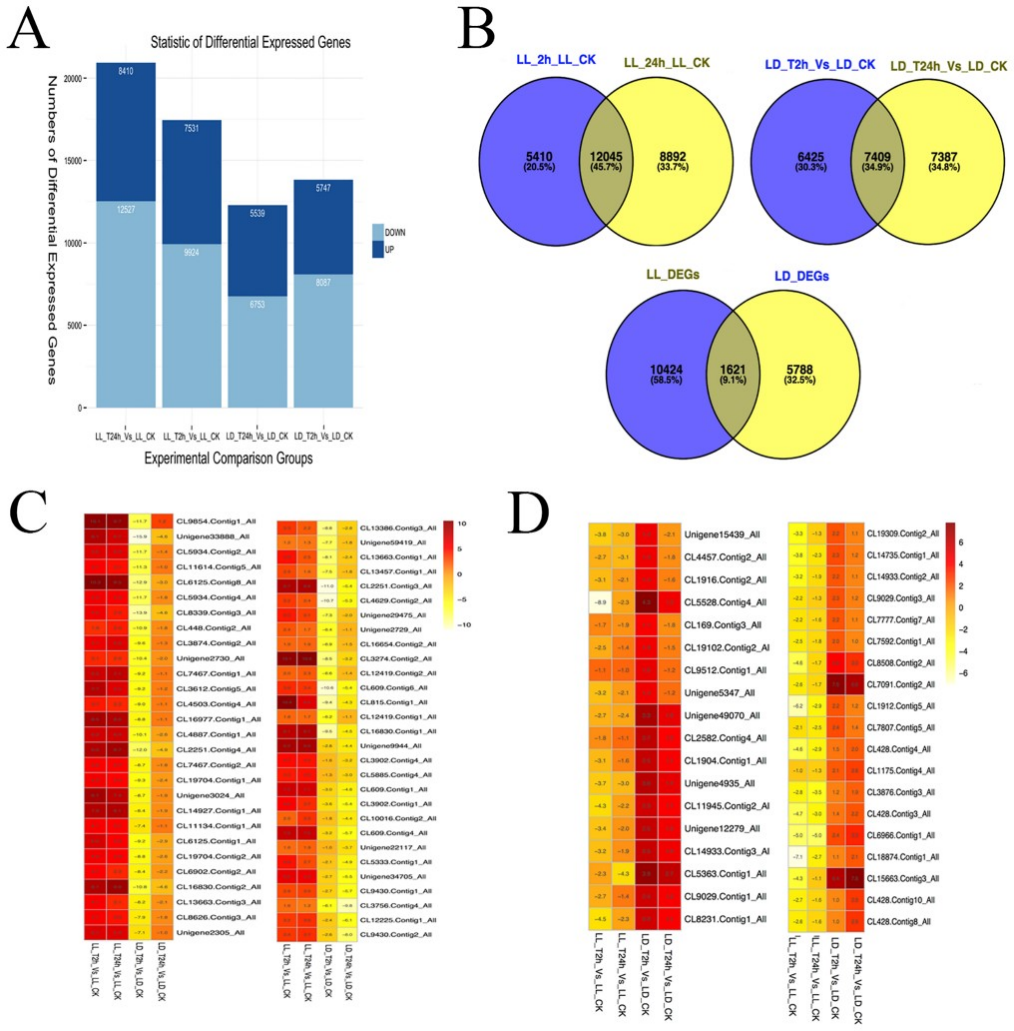
Differential expression genes (DEGs) associated with heat stress in lily. Fig 4A. Overview of up-regulated and down-regulated DEGs in *L*. *longiflorum* and *L*. *distichum*. Fig 4B. Co-modulated DEGs (common DEGs shared between *L*. *longiflorum* and *L*. *distichum)* identified in lily. Fig 4C. Specially DEGs selected with great alteration (up-regulated DEGs with significantly changed in *L*. *longiflorum* while down-regulated in *L*. *distichum*) identified in lily. Fig 4D. Specially DEGs selected with great alteration (Down-regulated DEGs with significantly changed in *L*. *longiflorum* while up-regulated in *L*. *distichum*) identified in lily.

To determine the differential expression genes (DEGs) among three stages of lilies in response to heat stress, we filtered the unigenes with an FDR ≤ 0.001 and |log2 (ratio)| ≥ 1 and found 26,347 DEGs in two of *L*. *longiflorum* libraries (LL_T2h vs. LL_CK and LL_T24h vs. LL_CK) and 21,221 DEGs in that of *L*. *distichum* libraries (LD_T2h vs. LD_CK and LD_T24h vs. LD_CK). Among these DEGs, 7,531 upregulated and 9,924 downregulated genes were detected in LL_T2h vs. LL_CK and 8,410 upregulated and 12,527 downregulated genes detected in LL_T24h vs. LL_CK. By contrast, 5,747 upregulated and 8,087 downregulated genes were detected in LD_T2h vs. LD_CK and 5,539 upregulated and 6,753 downregulated genes in LD_T24h vs. LD_CK (Fig 4A). The data suggested that the differentiation of genes between T24h and CK is larger than that between T2h and CK, and the number of DEGs of *L*. *longiflorum* was higher than that of *L*. *distichum* among the different heat stress stages. These findings indicate that, in *L*. *longiflorum*, transcript levels changed dramatically in contrast to that of *L*. *distichum* in response to heat stress, which was in accordance with the heat-resistance of *L*. *longiflorum* and *L*. *distichum*. These findings implied that our data was valuable for identifying heat-responsive genes and is therefore suitable for further analysis of the biological roles of these genes in lilies.

To further filter the DEGs between *L*. *longiflorum* and *L*. *distichum*, 1,621 common genes under high temperature were selected from LL_DEGs and LD_DEGs using an overlapping method (Fig 4B; S4 Table). Among these DEGs, we totally found 88 DEGs encoding for heat shock protein-like (HSPs) and 16 DEGs for Heat shock transcription factor (HSFs) (S5 Table). Of these HSPs, 21 and 16 DEGs were encoded for Class I heat shock protein-like and Class II heat shock protein-like, and 14, 11 and 13 DEGs encoded for heat shock 70 kDa protein, heat shock protein 83 and Small heat shock protein respectively. For the HSFs, DEGs mainly encoded for heat shock transcription factor A2, A3-like and B2c-like. Most these DEGs were upregulated in both lilies (*L*. *longiflorum* and *L*. *distichum*) under heat stress, indicating the similar behaviors of the HSPs and HSFs in other plant species when responsive to heat stress. In addition, 352 unigenes were obviously upregulated in *L*. *longiflorum* but downregulated in *L*. *distichum* during the heat-stress stages, and 133 unigenes in *L*. *longiflorum* showed decreased quantitative expression but increased transcript abundance in *L*. *distichum* (S6 Table). Furthermore, 57 upregulated DEGs with significant changes in *L*. *longiflorum* but downregulated in *L*. *distichum* are shown in Fig 4C and S7 Table, including glycine-rich cell wall structural protein-like (CL9854.Contig1_All), leucine-rich repeat receptor-like protein kinase *IMK2/IMK3* (Unigene2729_All, Unigene2730_All), caffeoyl-CoA O-methyltransferase-like (CL6125.Contig8_All), pathogenesis-related protein *PR10-5* (Unigene29475_All) and glutathione S-transferase U17/18/23-like (CL3902.Contig1_All, Unigene34705_All and CL4503.Contig4_All). In addition, 37 significantly downregulated genes in *L*. *longiflorum* and upregulated in *L*. *distichum* are shown in Fig 4D, such as 1-aminocyclopropane-1-carboxylate oxidase (CL428.Contig3/4/8/10_All), heat shock 70 kDa protein 8 (CL6966.Contig1_All), gibberellin-regulated protein 13-like (CL3876.Contig3_All), zinc finger protein 8-like (CL18874.Contig1_All) and signal peptide peptidase-like 2B (CL15663.Contig3_All). All the DEGs between *L*. *longiflorum* and *L*. *distichum* will be considered key candidate genes that are involved in the heat response and signalling pathways in lilies.

### Functional annotation of these common DEGs in lily

**Fig 5:**
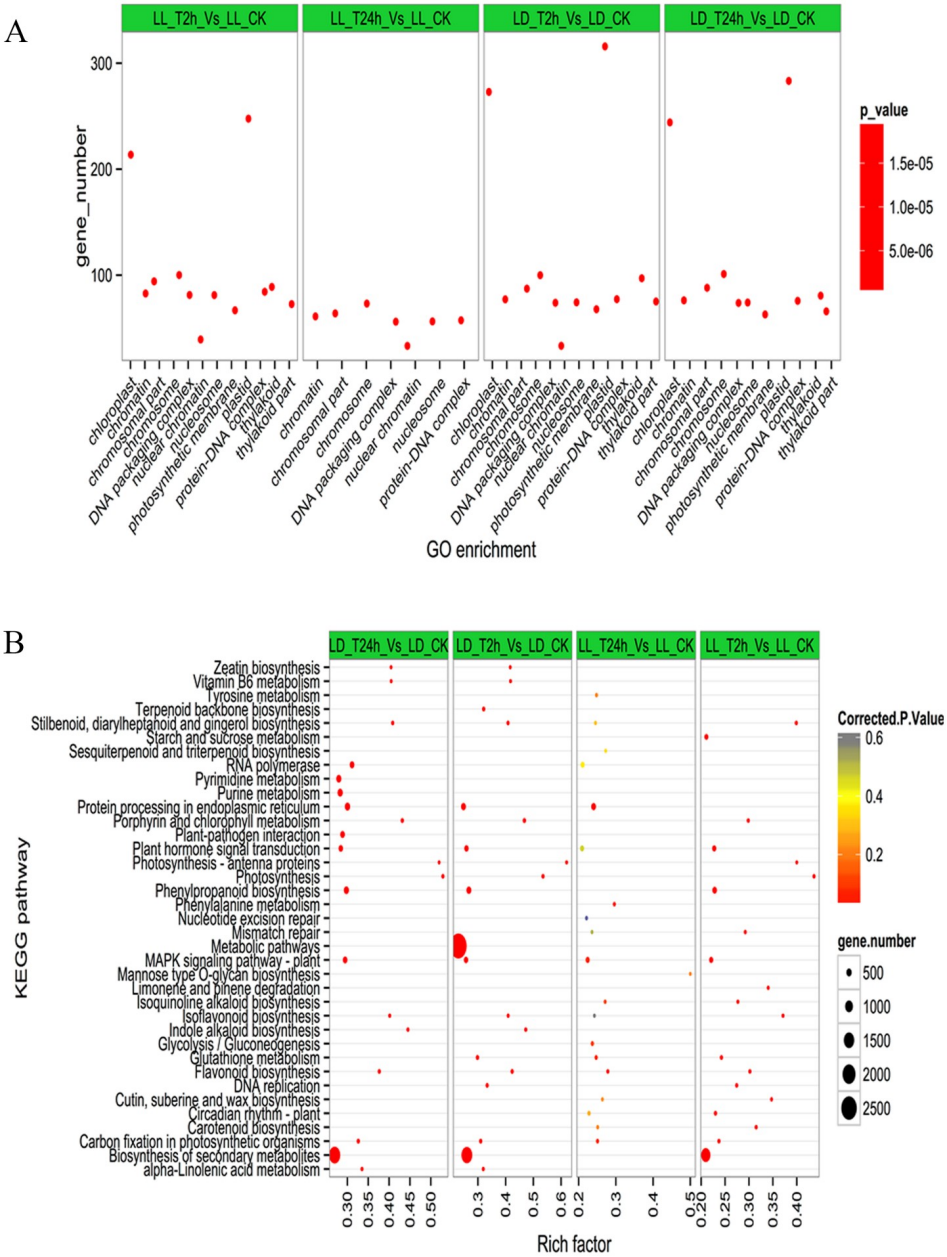
Functional enrichment analysis among differentially expressed genes (co-modulated DEGs) in lily. Fig 5A. Enriched GO terms of Co-modulated differentially expressed genes related to heat stress in Lily. GO terms are plotted on the ordinate, and the enrichment factor (rich factor) is plotted on the abscissa. The color of points represents the q-value, and the size of points represents the number of DEGs mapped to the reference pathway. Legends for the colour scale of q-values and size-scaling of the number of DEGs are shown to the right of the plot. Fig 5B. Pathway enrichment analysis among differentially expressed genes related to related to heat stress in Lily. Enriched KEGG pathway terms divided by different treatment time in *L*. *longiflorum* and *L*. *distichum* respectively. Red color indicates statically over represented.

To further identify these candidate genes responsive to short-term heat stress, we used gene ontology (GO) analysis to assign these DEGs of *L*. *longiflorum* and *L*. *distichum* to functional categories. DEGs that encoded genes in such categories as chromatin, chromosomal part, chromosome and protein-DNA complexes were enriched for *L*. *longiflorum* and that for chloroplast and plastid were greatly enriched during the heat-stress stages. In contrast, those DEGs for *L*. *distichum* were only enriched in the earlier stage of heat stress at 2 h, and a few DEGs were lost in the heat stress stage at 24 h, including chloroplast, plastid, thylakoid and thylakoid aspects (Fig 5A; S8 Table). These results were associated with the physiological or/and biochemistry changes of *L*. *distichum* seedings at 24 h under heat stress conditions.

The biological pathways influenced by heat stress were further evaluated by KEGG analysis of the DEGs in *L*. *longiflorum* and *L*. *distichum*. As shown in Fig 5B, a total of 24 pathways were affected by high temperature in *L*. *longiflorum*, while 27 pathways were responsive to high temperature in *L*. *distichum*, whereas the numbers of DEGs involved in these pathways of *L*. *longiflorum* far exceeded those of *L*. *distichum*. It should be noted that DEGs were enriched mainly in these pathways of *L*. *longiflorum*, such as RNA polymerase, Pyrimidine metabolism, Purine metabolism, Protein processing in endoplasmic reticulum, Plant-pathogen interaction, Plant hormone signal transduction, Metabolic pathways, Phenylpropanoid biosynthesis, and Biosynthesis of secondary metabolites. In contrast, these pathways were not evident in *L*. *distichum* when exposed to one or two heat stress stages, which suggested that these pathways probably play a key role in regulating the short-term heat response in lilies. Specifically, 417 genes in protein processing in endoplasmic reticulum and 2,702 genes in metabolic pathways showed strongly changed expression under heat stress for 2 h in *L*. *longiflorum* in contrast to that in *L*. *distichum* (S9 Table). In addition, approximately 500 genes in each pathway of pyrimidine metabolism, purine metabolism, plant-pathogen interaction, plant hormone signal transduction, and phenylpropanoid biosynthesis and approximately 1,500 genes in biosynthesis of secondary metabolites were also affected under heat stress for 24 h in *L*. *longiflorum* but not in *L*. *distichum* (S9 Table). The collected data revealed the changes in molecular function and signalling pathways in lilies responding to heat stress as well as the differences between the heat-tolerant *L*. *longiflorum* and the heat-sensitive *L*. *distichum*.

### Identification of candidate heat responsive DEGs by qRT-PCR in lily

**Fig 6:**
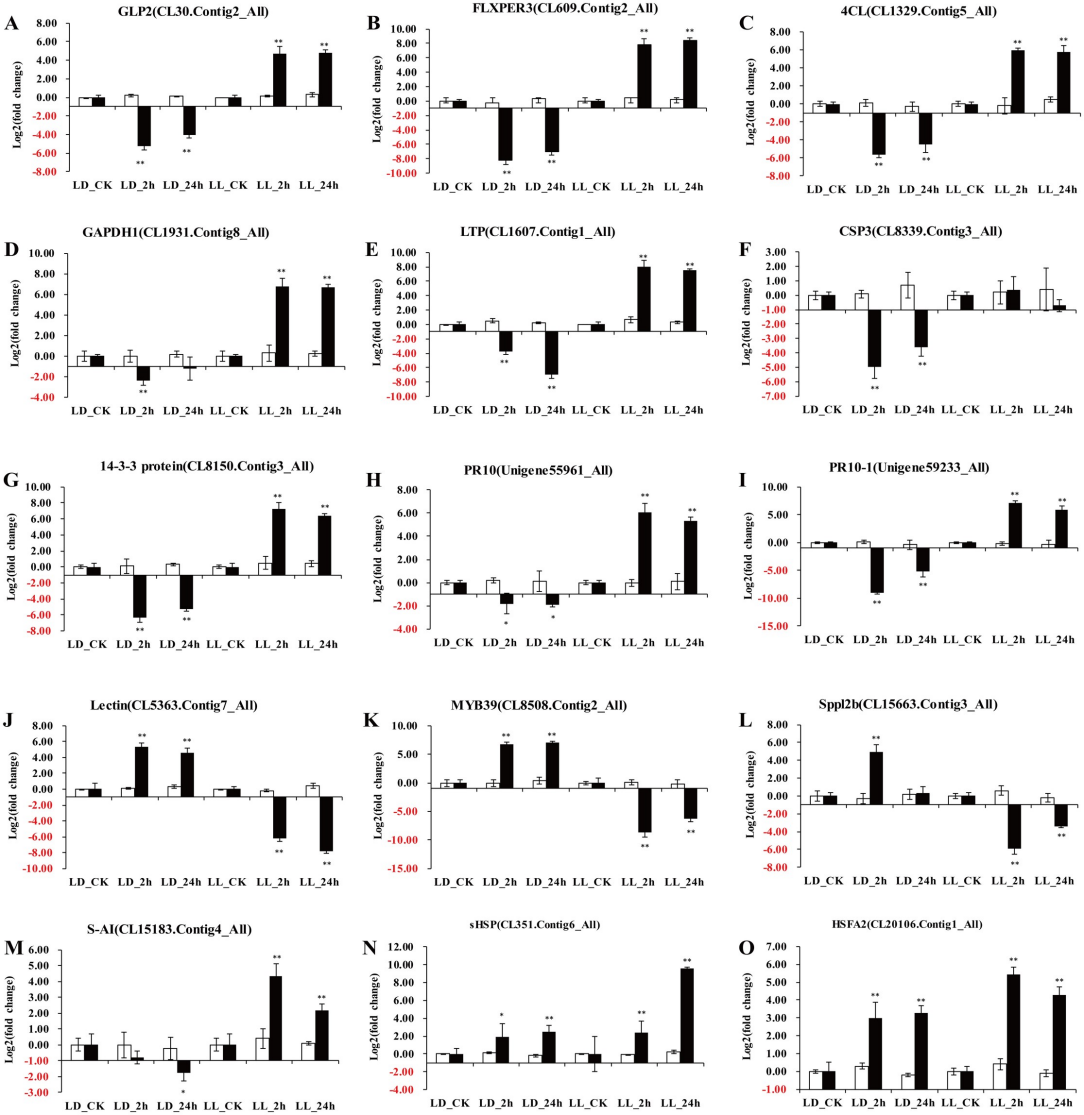
The quantitative real-time polymerase chain reaction (qRT-PCR) analysis of expression levels of 15 selected genes under different heat stress treatment time in *L*. *longiflorum* and *L*. *distichum*. Three independent biological repeats were performed, and all data points were the means of three biological replicates ± standard error (SE). A student’s t-test was used to calculate the significance of differences (*: P < 0.05, **: P < 0.01).

**Fig 7:**
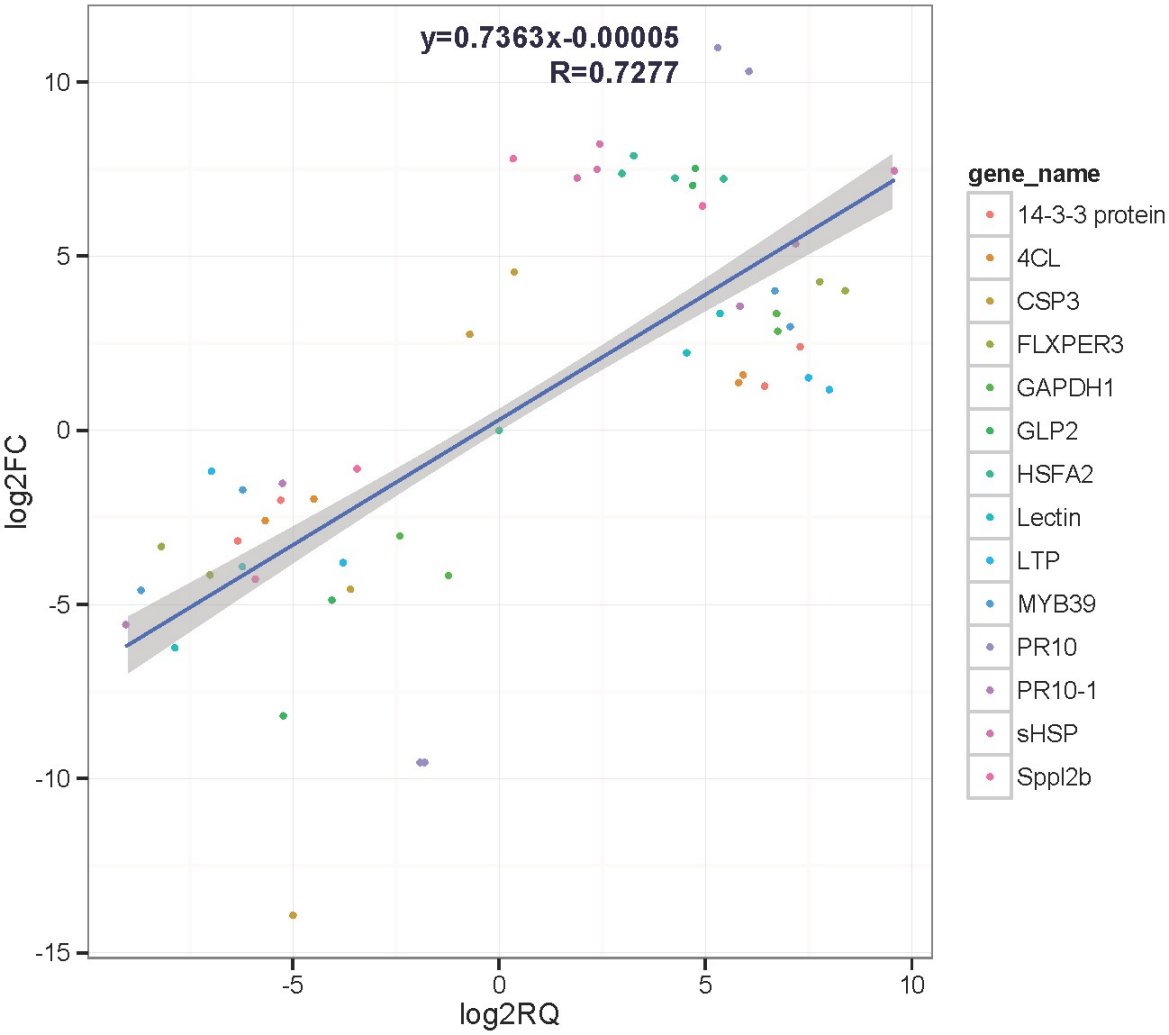
Correlations of transcript levels of heat response genes in *L*. *longiflorum* and *L*. *distichum* between RNA-seq and qPCR data.

To validate the DEGs obtained from RNA sequencing, a total of 15 DEGs showing diverse expression profiles during the heat stress stages were chosen to perform the qRT-PCR analysis. The expression levels of 9 putative DEGs, such as CL30.Contig2_All (*GLP2*), CL609.Contig2_All (*FLXPER3*), CL1329.Contig5_All (*4CL*), CL1607.Contig1_All (*LTP*), CL1931.Contig8_All (*GAPDH1*), CL8150.Contig3_All (14-3-3 protein), CL8339.Contig3_All (*CSP3*), Unigene55961_All (*PR10*) and Unigene59233_All (*PR10-1*), which were upregulated in *L*. *longiflorum* but downregulated in *L*. *distichum* at the heat-treatment stages, were further confirmed, except for the *CSP3* gene, which displayed a similar expression between CK and heat treatment at 2 h or 24 h based on the qRT-PCR results (Fig 6). In addition, the qRT-PCR results showed 3 putative DEGs, CL5363.Contig7_All (Lectin), CL8508.Contig2_All (*MYB39*), and CL15663.Contig3_All (*Sppl2b*), which were downregulated in *L*. *longiflorum* and upregulated in *L*. *distichum* at the heat-treatment stages and 2 putative DEGs, CL351.Contig6_All (*sHSP*) and CL20106.Contig1_All (*HSFA2*), which were highly induced expressed in *L*. *longiflorum* and *L*. *distichum* at the heat-treatment stages, showing consistent results with the RNA-Seq expression data. Correlation between the RNA-seq and qPCR data were conducted, which indicated that both results were basically consistent for gene expression profiles (Fig 7, r = 0.7277). Althouth the expression of CL15183.Contig4_All (S-AI) was induced in *L*. *longiflorum* and repressed in *L*. *distichum*, which was a different result from that obtained using RNA-seq (Fig 6). The above results further confirmed a high reliability of the RNA-seq data obtained in our study.

## Discussion

High temperature is a strong barrier for lily growth and development and results in a poor quality of lily flowers and reduced bulb yield. As a result, it will be critical to confer lily species that can withstand high temperatures. Despite the physiological analysis and studies of heat shock transcription factor *HSFs* and heat shock protein HSPs on heat stress in lilies [13, 14, 17], the underlying molecular mechanisms remain unclear. In the present study, comparative transcriptome analysis was performed for lily response to high temperature for different stages using RNA-seq. Because the genomic database of lily is not yet available, *de novo* transcriptome sequencing was performed, referring to previous studies [19, 39]. Comparing the transcriptomes of the heat-tolerant *L*. *longiflorum* and the heat-sensitive *L*. *distichum*, differentially expressed genes and signalling pathways during heat stress were identified in lily plants.

In previous studies, the physiological changes of lilies responding to high temperature had been analysed, including the photosynthetic pigment content, proline content, electrolyte leakage and MDA concentration, root activity and antioxidant enzyme activities at several levels of heat stress (31°C, 34°C, 37°C, 39°C, 42°C, 47°C) [13, 40, 41]. In most places in China, maximum temperatures can reach 42°C in the summer. Therefore, to uncover the optimal time-point of lily exposure to heat for RNA-seq, we performed the physiological analysis under the 42°C heat treatment for several hours. The results showed that the soluble sugar content was significantly upregulated, and the chlorophyll content obviously reduced, when lily seedlings were exposed to heat stress. The increase in carbohydrates was paralleled by the proline content in *L*. *longiflorum* leaves (Fig 2C–2E), suggesting that these components in lilies may act synergistically under high temperature stress. We also found that lily seedlings at two heat stress stages (2 h and 24 h) exhibited a clear transition of morphological or/and physiological responses. In addition, the heat-tolerant variety (*L*. *longiflorum*) suffered delayed membrane damage and colour change compared with the heat-sensitive variety (*L*. *distichum*) (Figs 1 and 2), which was similar to the heat response in other plants, such as in peppers, rices and cucumbers [42–44]. Besides that, the heat-tolerant *L*. *longiflorum* presented no symptom of wilting whereas heat-sensitive *L*. *distichum* showed signs of wilting under heat stress for 24h. The wilt phenotypes were observed in the heat-susceptible cultivar S590 but not in the heat-tolerant R597 cultivar. In total, samples of lily seedlings exposed to high temperature for 2 h and 24 h and a control (CK) were selected for the following analyses.

Our study of the transcriptome analysis by RNA-seq were based on the BGISEQ-500 platform of BGI’ tech, using paired-end sequencing technology to produce a large-scale EST database. Approximately 39.55 Gb of data were generated and assembled into 130,170 unigenes with a mean length of 875 bp. The average length in our study was similar to that of a published research in *L*. *lancifolium* [19]. Of all the unigenes, 34,301 (26.4%) unigenes could match homologues in NR, InterPro, SwissProt, COG and KEGG database (Fig 3C), revealing that the numbers of transcripts were represented in the well-known data. These genes were performed for GO classification and predicted to primarily function in metabolic process, cellular process, cell and cell part, catalytic activity and binding (S2A Fig), which were similar to the findings in the heat response of pepper, rice and grape [42, 45, 46]. This finding also suggested the complexity of the high temperature signal transcription in Lilium. Statistical analysis of differentially expressed genes (DEGs) indicated that the changes in gene expression in *L*. *longiflorum* were larger than those in *L*. *distichum*, whether at 2 h or 24 h of heat stress, suggesting that more genes involved in heat-tolerance might be expressed in the heat-tolerant *L*. *longiflorum* than in the heat-sensitive *L*. *distichum* during heat stress (Fig 4A and 4B), including genes in Plant-pathogen interaction and Metabolic pathways, etc. The results were partly similar to those in Chinese cabbage [21] and Pepper [42]. Out of 5410+12045+8890 DEGs in *L*. *longiflorum* and 6425+7409+7387 DEGs in *L*. *distichum* (Fig 4B), 12045 and 7409 candidate genes in *L*. *longiflorum* and *L*. *distichum* were used for KEGG pathway analysis, respectively. In our results, genes were differently expressed in these pathways including Zeatin biosynthesis, Vitamin B6 metabolism, Terpenoid backbone biosynthesis, Indole alkaloid biosynthesis and Alpha-Linolenic acid metabolism and significantly modulated in Protein processing in endoplasmic reticulum and Metabolic pathways at the heat stress for 2 h in *L*. *longiflorum* compared with *L*. *distichum*, suggesting that genes in the latter two pathways might play important roles in regulating the early heat response of lilies. In addition, these pathways comprised of RNA polymerase, Pyrimidine metabolism, Purine metabolism, Plant-pathogen interaction and Plant hormone signal transduction were highly enriched in *L*. *longiflorum* more than that in *L*. *distichum* exposure to high temperature for 24 h, suggesting their critical roles in heat-tolerance of lilies responding to high temperature. Notably, *L*. *longiflorum* genes were predicted to function mainly in metabolic pathways, biosynthesis of secondary metabolites, and protein processing in endoplasmic reticulum, which was similar to the data of previous studies [22, 42, 47].

### HSF-HSP signalling pathway in lily heat response

It was well-documented that the expression of heat shock transcription factor HSFs and heat shock protein HSPs were obviously induced when plants such as *Tomato* and *wheat* etc. are exposed to elevated temperature [22, 23, 48–50]. HSFs function as central components in plant response to heat stress by regulating the expression of HSPs and other chaperones [12]. In the present data, a total of 16 DEGs encoding for HSFs and 87 DEGs for HSPs were significantly upregulated in both lilies (*L*. *longiflorum* and *L*. *distichum*), taking the 0.9870% and 5.3671% in total of these DEGs. The findings further identified our sequencing data of lilies exposure to heat stress. These DEGs such as heat shock transcription factor A2 (Unigene14710_All, Unigene14708_All, CL209.Contig1_All and CL20106.Contig1,3,4,6_All), A3 (CL5951.Contig2,5_All), small heat shock protein (Unigene22366_All, Unigene22367_All and CL351.Contig6_All), heat shock protein 83 (CL20541.Contig9_All), class II heat shock protein-like (CL7583.Contig1_All, CL163.Contig4_All and CL16968.Contig1_All), and Hsp70-Hsp90 organizing protein-like (CL2253.Contig2,3,4,6,10,11,12_All), as well as Hsp70-binding protein 1-like (CL1733.Contig3,5,6,7_All), was in agreement with previous studies and further confirmed the conserved mechanism of heat response in *Lilium* and other plants [3, 23, 51–53]. Moreover, heat shock transcription factor HSFA2, HSFA3 from *L*. *longiflorum* has been indicated an important role in the heat signaling pathway by reverse genetic transformation in *Arabidopsis* [14, 16].

### Plant hormone signal transduction and lily heat response

Auxin, abscisic acid (ABA), and salicylic acid (SA) have been shown to be important in the heat stress response of various plants and may possibly regulate heat stress signalling pathways [3]. In our data, the candidate genes in auxin response signalling pathway were obviously decreased by heat stress in heat-sensitive *L*. *distichum*, including *TIR1*, *Aux/IAA*, *ARF* and *GH3* genes, which was consistent with previous studies [54, 55]. However, these genes corresponding to *TIR1*, *Aux/IAA* and *ARF* remained obscure in the heat-tolerant *L*. *longiflorum*, specifically, the *GH3* genes (Unigene12571_All, CL6902.Contig2_All, CL6902.Contig1_All) were upregulated in the heat-tolerant genotype, suggesting that *GH3* was possibly involved in heat tolerance. Previous studies indicated that ABA signalling is upregulated by heat stress in pea [56] and *Agrostis stolonifera* [57], and the Pyrabactin resistance (*PYR*)/*PYR1-like* (PYL) genes were upregulated after heat stress in the heat-susceptible pepper [42]. However, in the present study, the *PYR/PYL* genes of ABA receptor (CL14565.Contig1-5_All, CL20275.Contig1_All, CL8078.Contig1_All, CL20508.Contig6_All, Unigene14869_All, CL762.Contig8_All, CL8513.Contig1_All) were repressed in heat-sensitive *L*. *distichum*. In addition, ABA responses negatively regulated by the type 2C protein phosphatases (*PP2C*) the and the candidate genes of *PP2C* were upregulated in heat-sensitive *L*. *distichum*, indicating that ABA signalling might possibly be repressed and that ABA signalling is critical in the response of lilies to high temperature. SA can improve antioxidant capacity and enhance heat resistance in rice [58] and Arabidopsis [59]; we also found target genes of *NPR1* and TGA involved in SA metabolic pathway were obviously upregulated in heat-tolerant *L*. *longiflorum* after heat stress (2 h and 24 h) compared with being downregulated in heat-sensitive *L*. *distichum*, which suggested that *NPR1* (CL9114.Contig1-4_All, and Unigene589_All) and *TGA* (CL925.Contig1-5_All, CL14874.Contig1-2_All, CL910.Contig1_All, Unigene19378_All, Unigene22208_All, CL11558.Contig5-6_All, CL11449.Contig2_All) might be promising targets for the improvement of lily heat resistance and confirmed the critical role of SA response to heat stress.

### Plant-pathogen interaction and phenylpropanoid/lignin biosynthesis in lily heat response

Specifically, we found pathogenesis-related protein 10 (*PR10*) genes, such as *PR10-1* (Unigene59233_All, CL523.Contig6_All), *PR10-2* (Unigene7708_All), *PR10-3* (CL20508.Contig3_All, CL20508.Contig7_All, CL20508.Contig9-11_All, Unigene19673_All, Unigene29327_All, CL20508.Contig2_All), *PR10-4* (Unigene52606_All, CL20508.Contig6_All, CL20508.Contig8_All), *PR10-5* (Unigene29475_All), *PR10-6* (Unigene52945_All), *PR10-7* (CL523.Contig1_All), *PR10-8* (CL20508.Contig5_All) and *PR10* (CL4502.Contig2-3_All), glutathione S-transferase genes (Unigene34705_All, CL3902.Contig1,2,4,6,7_All, CL651.Contig3,7_All, CL4503.Contig4_All, CL3718.Contig3_All, CL863.Contig7,8_All, CL15152.Contig3_All, CL1001.Contig4_All), 14-3-3 protein gene (CL8150.Contig3_All) and germin-like protein GLP1,2 (CL3274.Contig2_All, CL30.Contig2_All) as well as WRKY-related gene (CL170.Contig3_All, CL3728.Contig1,8_All), involved in Plant-pathogen interaction were surprisingly upregulated in heat-tolerant *L*. *longiflorum* but repressed in heat-sensitive *L*. *distichum* after heat stress for 2 h and 24 h [60–62]. These findings are very interesting and are similar to the finding that immune response genes were more expressed in the heat-tolerant Chinese cabbage ‘XK’ after 12 h of heat treatment [21]. In addition, genes such has *4CL* (CL1329.Contig5_All), *CCoAOMT* (CL6125.Contig1, 2,4,5,8_All), ferulate-5-hydroxylase (F5H, CL12000.Contig1_All), and peroxidase (CL609.Contig1, 4, 6_All, CL10016.Contig2_All, CL9430.Contig1,2_All), which are involved in the Phenylpropanoid/ monolignol biosynthesis pathway, were also upregulated in *L*. *longiflorum* but downregulated in *L*. *distichum*. This finding first indicates that lignin biosynthesis is related to heat stress and might play an important role in lily plant response to high temperature.

### Kinase signalling genes in lily heat response

Kinases are critical components involved in signal transduction pathway in plant response to various environmental stimuli. Of these, redox-sensitive transcription factors are activated by the *MAPK* signalling pathway, including the *Zat* family, the *WRKY* family, *MBF1c* and *Rboh* [3], and has been involved in a heat shock response of alfalfa cells [63, 64]. Similarly, in our results, the candidate genes *MPK4* (CL15793.Contig2_All) and *NDPK2* (CL7963.Contig2, 3_All) in heat-tolerant *L*. *longiflorum* were upregulated, while they (CL15793.Contig1, 4_All and CL15981.Contig1, 2_All) were repressed in heat-sensitive *L*. *distichum* after 24 h of heat stress. In addition, *MKK3* (CL6477.Contig3, 4_All) was induced by heat stress in *L*. *longiflorum*, although it showed no response in *L*. *distichum*. Other kinases, such as leucine-rich repeat receptor-like protein kinase (*IMK2*, Unigene2729_All; *IMK3*, Unigene2730_All, CL5334.Contig5_All), proline-rich receptor-like protein kinase *PERK1* (CL5771.Contig2_All), CBL-interacting serine/threonine-protein kinase 23-like (Unigene45_All, CL20344.Contig3_All), guanylate kinase GK-1 (CL6633.Contig5_All), ATP-dependent 6-phosphofructokinase 2-like (CL11999.Contig4_All), mitogen-activated protein kinase *NPK1-like* (CL3282.Contig2_All), and ribose-phosphate pyrophosphokinase 1-like (CL5046.Contig1_All), were also upregulated in *L*. *longiflorum* but downregulated in *L*. *distichum*, which suggested that they could play important roles in lily heat-response pathways. These data further provide novel insights into the idea that various kinases are involved in the heat response signalling pathway of lilies and might be used as key targets for genetic modification of lilies.

### Calcium and phospholipid pathways in lily heat response

Previous studies have indicated that calcium signalling plays dynamic and key roles in the regulation of heat-responsive genes [65, 66]. Calcium-binding protein *CML19* (CL5289.Contig1_All) was induced during heat stress in the two genotypes, and calmodulin (*CALM*, CL1722.Contig4_All) was upregulated after 24 h of heat stress in *L*. *longiflorum* and downregulated in *L*. *distichum*. The phospholipid-based signalling is involved in response to various abiotic and biotic stresses, including heat stress [67]. Under heat stress, the G protein transduces the heat-initiated signal required for *PIP2* and *PA* accumulation involved in Phospholipid pathway [68]. In addition, phosphatidylinositol phospholipase C (*PLC*) further catalysed the hydrolysis of *PIP2* to produce *DAG* and *IP3*, which is rapidly converted to *IP6* for the release of Ca^2+^ [69]. In the present study, candidate genes encoding for G1 protein-like (CL5934.Contig2, 4_All, CL8626.Contig3_All, and CL11614.Contig5_All) were significantly upregulated in heat-tolerant *L*. *longiflorum* and downregulated in heat-sensitive *L*. *distichum*. In addition, *PLC* (CL8354.Contig2_All, CL8354.Contig3_All, CL14032.Contig2_All) was also induced in the heat-tolerant genotype, which indicated that the two genes might be responsible for the heat-stress resistance.

## Conclusion

To further uncover the molecular mechanisms of *Lilium* plant response to heat stress, future work should select specific genes and validate their biological functions by reverse genetics and protein-to-protein interactions. In conclusion, the present study is the first to determine the physiological or/and biochemical changes of a thermo-tolerance lily flower *L*. *longiflorum* and a thermo-sensitive lily flower *L*. *distichum* under short-term heat stress and to identify the heat-response signalling pathways and differentially expressed genes using comparative RNA-seq analysis. Approximately 1,621 significantly modulated genes were involved in the two lilies, of which 352 genes were upregulated in the heat-tolerant *L*. *longiflorum* but suppressed in the heat-sensitive *L*. *distichum*. These genes were mainly involved in metabolic pathways, phenylpropanoid biosynthesis, plant-pathogen interactions, plant hormone signal transduction, and kinase signalling pathways. The collected data provide new insights into the molecular signalling networks of lilies during heat tolerance and may facilitate the heat-resistance breeding of lily species by genetic modification approaches.

## Supporting information

S1 Fig Information of BUSCO assemble for assembled transcripts by TRINITY. (PNG)

S2 Fig Functional annotation and classification of the assembled unigenes from the lily transcriptome. S2A Fig. Gene ontology classification of the unigenes from the lily transcriptome under heat stress. S2B Fig. COG function classification of unigenes from the lily transcriptome under heat stress. S2C Fig. KEGG pathway annotation of assembled unigenes from the lily transcriptome under heat stress.
(PNG)

S3 Fig Clustering and heat map of global expression genes based on the RPKM value in the heat-tolerant *L*. *longiflorum* and the heat-sensitive *L*. *distichum*. (PNG)

S1 Table List of primers used for qRT-PCR analysis of candidate DEGs related to heat stress in lily. (XLSX)

S2 Table Clean reads were generated from *L*. *longiflorum* and *L*. *distichum*. (XLSX)

S3 Table The abundance of globally expressed genes identified using FRKM under heat stress in *L*. *longiflorum* and *L*. *distichum*. (XLSX)

S4 Table Global profiles of the expression of genes related to heat stress identified in lily. (XLSX)

S5 Table DEGs encoding HSPs and HSFs related to heat stress identified in lily. (XLSX)

S6 Table Expression levels of common DEGs related to heat stress identified in lily. (XLSX)

S7 Table 57 upregulated DEGs with significant changes in *L*. *longiflorum* but downregulated in *L*. *distichum*. (XLSX)

S8 Table GO annotation of common DEGs related to heat stress identified in lily. (XLSX)

S9 Table KEGG pathway enrichment of common DEGs related to heat stress identified in lily. (XLSX)

## Abbreviations

BP: Biological process
CC: Cellular component
COG: Clusters of Orthologous Groups
DEGs: Differential expression genes
FPKM: Fragments per kilobase of transcript per million mapped reads
GO: Gene ontology
InterPro: Inter-Pro proteins
KEGG: Kyoto encyclopedia of genes and genomes
LD: Lilium distichum
LL: Lilium longiflorum
MF: Molecular function
NCBI: National center for biotechnology information
NR: Non-redundant protein
SwissProt: Swiss-Prot proteins
