# Supplementary figures and images for: Comparative transcriptome analysis reveals heat stress-responsive genes and their signalling pathways in lilies (Lilium longiflorum vs. Lilium distichum)
Source: PLoS One. 2020 Oct 2;15(10):e0239605. doi: 10.1371/journal.pone.0239605 (PMC7531851; doi:10.1371/journal.pone.0239605)

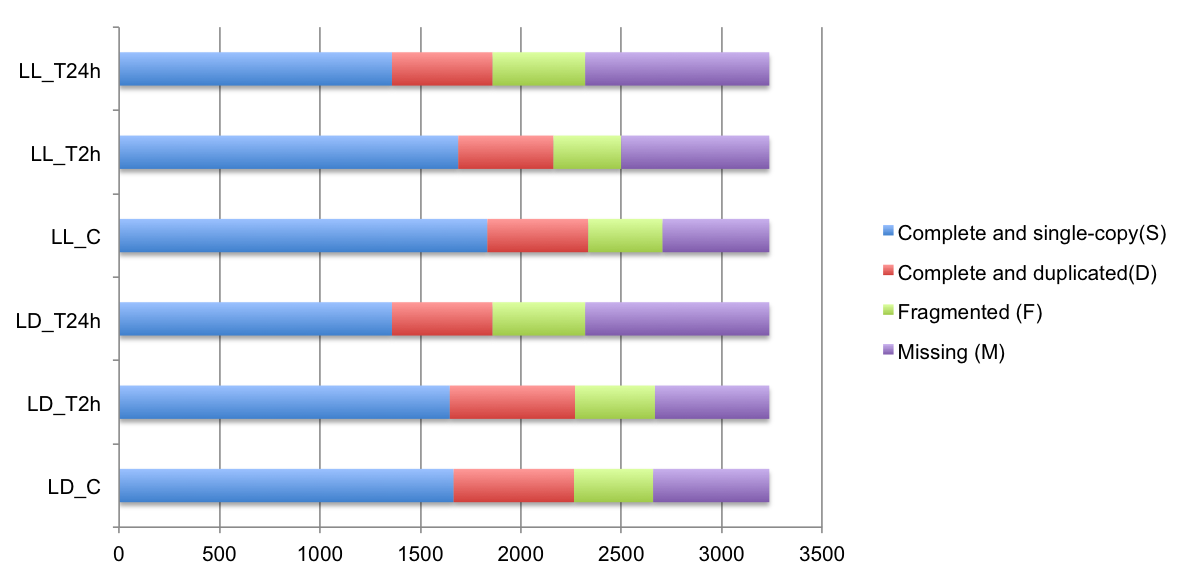

Supplement: S1 Fig — (PNG) [file pone.0239605.s001.png]

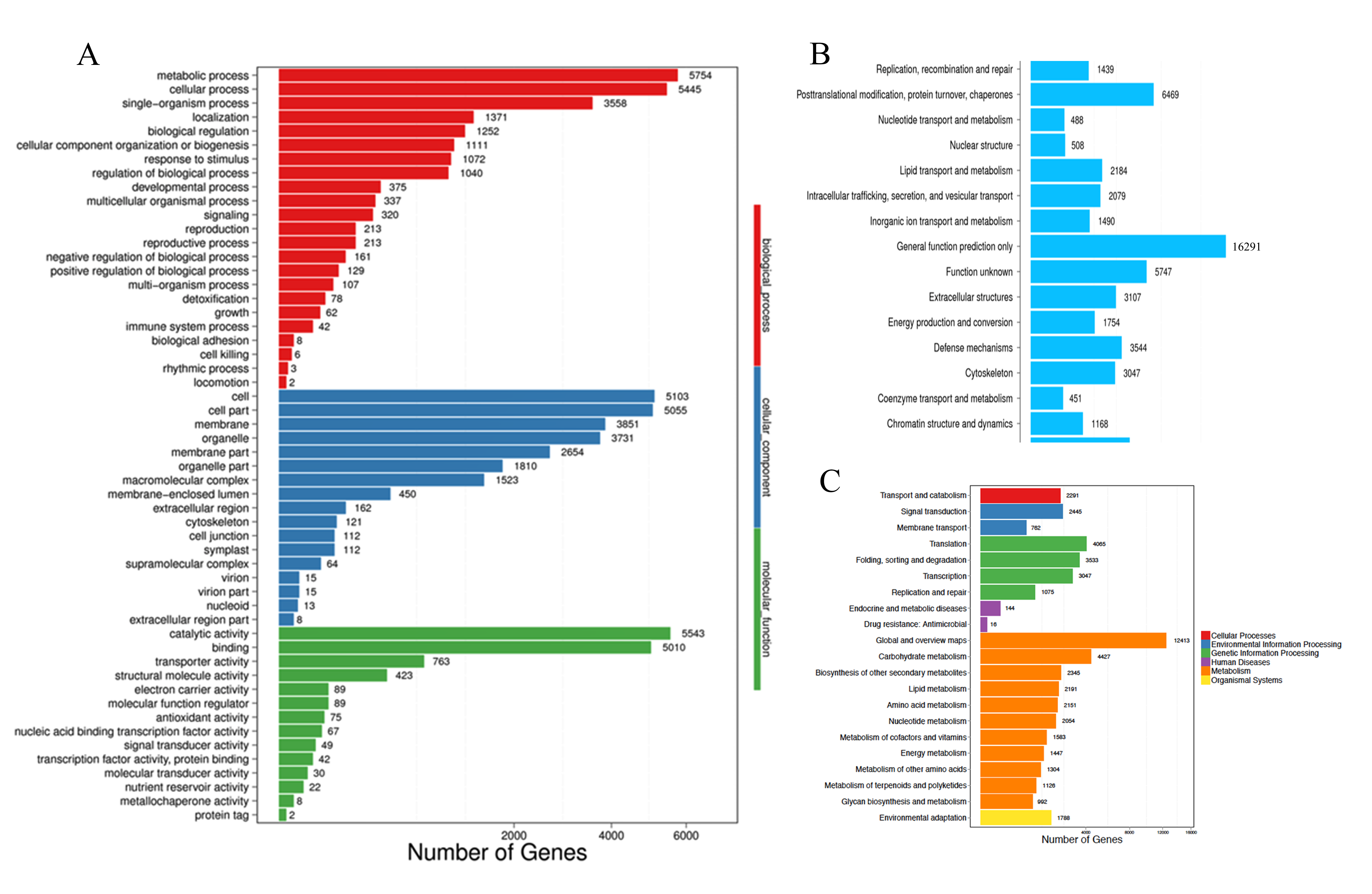

Supplement: S2 Fig — S2A Fig. Gene ontology classification of the unigenes from the lily transcriptome under heat stress. S2B Fig. COG function classification of unigenes from the lily transcriptome under heat stress. S2C Fig. KEGG pathway annotation of assembled unigenes from the lily transcriptome under heat stress. (PNG) [file pone.0239605.s002.png]

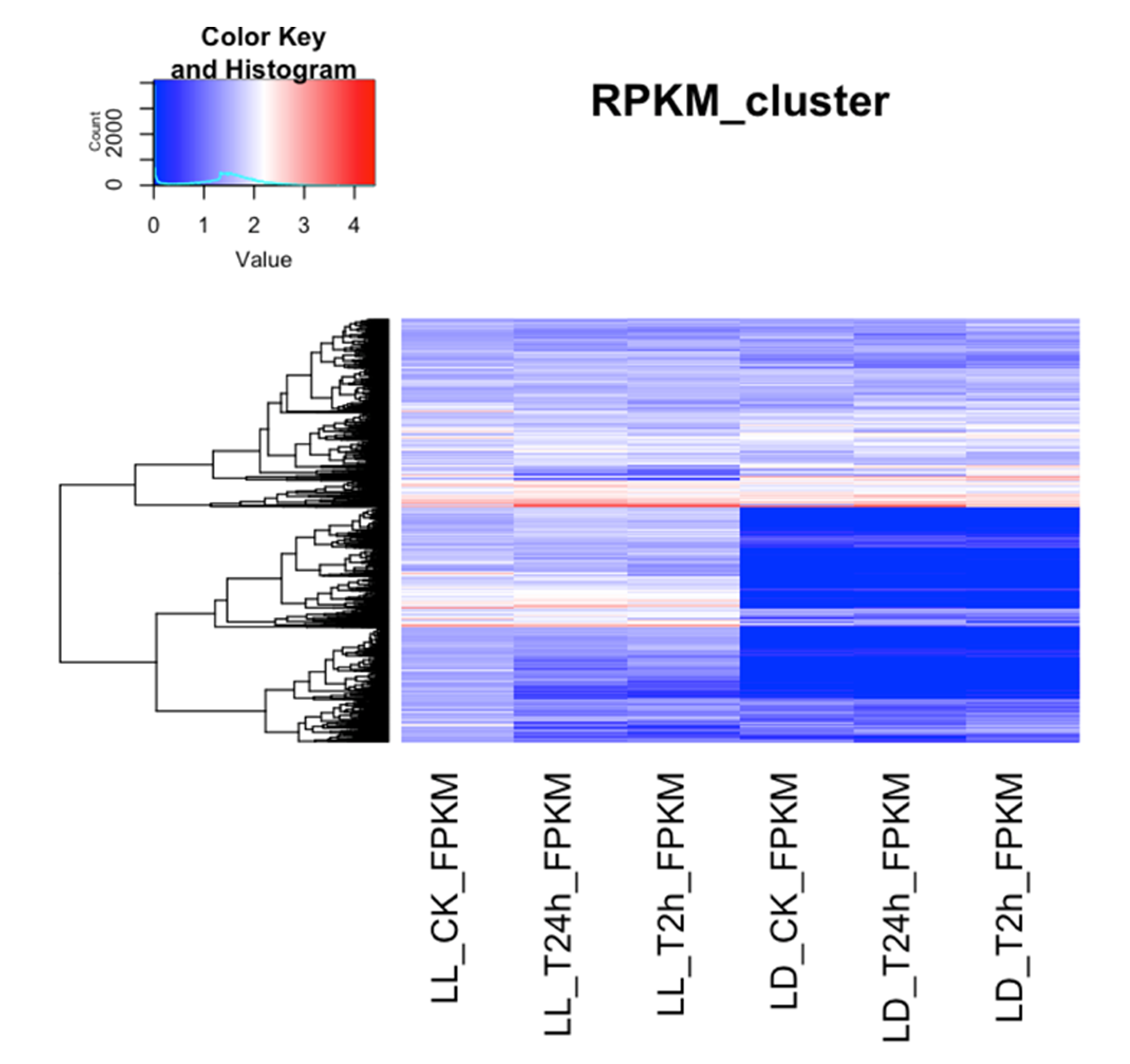

Supplement: S3 Fig — (PNG) [file pone.0239605.s003.png]
